# Pilot Scale Hybrid Organic/Inorganic Coatings on a Polyolefin Separator to Enhance Dimensional Stability for Thermally Stable Long-Life Rechargeable Batteries

**DOI:** 10.3390/polym14214474

**Published:** 2022-10-22

**Authors:** Hyoungwoo Choi, Byoung-Sun Lee

**Affiliations:** 1 Samsung Advanced Institute of Technology, Suwon 16678, Korea; 2 Department of Fiber Convergence Materials Engineering, School of Polymer System, Dankook University, Yongin 16890, Korea

**Keywords:** crosslinkable polyamide-imide, hybrid organic/inorganic coating, pilot-scale, mini-18650 Li-ion cell, thermal stability

## Abstract

The electric vehicle and energy storage markets have grown rapidly in recent years. Thermal runaway caused by malfunctioning Li-ion batteries is an urgent issue with many causes (e.g., mechanical, electrical, and thermal abuse). The most common cause of thermal runaway is the formation of an internal short circuit because of damage to the separator. There has been significant effort to improve the design of separators, but to our knowledge, only inorganic nanoparticle coatings are used in commercial Li-ion batteries. Here, hybrid organic/inorganic coating layers are synthesized in a pilot-scale process that was developed from a crosslinkable polyamide-imide synthesis technique. The fabrication process is optimized to achieve reproducible hybrid organic/inorganic coating layers that are thin (≤4 μm), permeable (≤250 s/100 cc), and thermally stable beyond 150 °C. The hybrid coating layer is applied to mini-18650 Li-ion cells to show that the discharge capacity did not change at low discharge rates, and the retention capacity after 500 cycles was better than that of the reference cells used for comparison. This work demonstrates that a novel hybrid coating layer has the potential to improve the stability of commercial Li-ion batteries.

## 1. Introduction

Li-ion batteries play a crucial role in modern daily life as a robust portable power source with high energy density [1], small memory effect [2], and long lifespan [3]. Consumer use of “smart” devices has increased substantially as battery energy density increases, at a rate of 5.5 Wh kg^−1^ per year [4], and the availability of large batteries capable of storing 60 kWh has contributed to the growth of environmentally friendly electric vehicles [5]. Furthermore, smart grids reduce carbon emissions by generating electricity from solar, wind, geothermal, and tidal energy sources and storing it in and supplying it from energy storage systems [6]. Development of the next generation rechargeable batteries, such as Zn based batteries [7,8,9], has been raising expectations for far safer and much more advanced energy storage devices that contribute to smarter and cleaner lives.

Li-ion batteries are an integrated component in a wide range of technologies; however, malfunctioning batteries are susceptible to thermal runaway, which presents a significant risk to users and their property. Thermal runaway can be caused by mechanical, electric, and thermal abuse [10]. Mechanical abuse typically results in deformation, while electrical abuse encourages the growth of dendrites and elevated temperatures, which can cause thermal damage. Any damage to the battery separators can result in electrical short circuits, which are the most direct cause of thermal runaway [11]. Once an internal short circuit is formed, exothermic reactions, such as solid electrolyte interface decomposition, separator disintegration, and redox reactions between the cathode and anode are initiated, the battery temperature increases rapidly [12,13]. Therefore, it is important that improvements be made to separator designs to prevent or delay damage and improve the safety of Li-ion batteries.

Research efforts have been focused on the development of advanced separators that address the aforementioned issues. These include: separators fabricated from phase-changing thermoregulating coaxial fibers [14], flame-retardant coaxial fibers [15], and an electrochemically active silica sandwich [16]. Among the technologies that have been explored, separators coated with inorganic nanoparticles are of particular interest as they are low-cost and simple to process, both of which are attractive properties for commercial production. Electrochemically inactive inorganic materials, such as SiO_2_ [17,18,19], Al_2_O_3_ [20,21,22], and AlOOH [23,24,25], have been used to improve the thermal and mechanical performance of separators, and increase their wettability at the interface with the electrolyte. Polyvinyl alcohol [24], polyvinylidene hexafluoropropylene [21], and polyurethane acrylate [26] have been used as binders. Most studies concluded that inorganic nanoparticle-coated separators have superior electrolyte wettability [20], thermally stability [17], and improved electrochemical performances [18], but research has focused on their use in single coin cells and small pouch cells. Therefore, there is a need to develop inorganic nanoparticle separators suitable for the fabrication of large-scale Li-ion batteries. Furthermore, the inorganic nanoparticles and organic components must be thermally stable. In this work, we design a thermally stable organic crosslinked polyamide-imide component and optimize a pilot-scale process to apply a hybrid organic/inorganic coating to a polyolefin separator. The consequences of applying the hybrid layer are thoroughly evaluated by testing its electrochemical performance in mini-18650 Li-ion cells.

## 2. Experimental

### 2.1. Crosslinkable Polyamide-Imide Preparation

Hexafluoroisopropylidene-bis-phthalic anhydride (6-FDA; Sigma-Aldrich, Burlington, MA, USA), toluene diisocyanate (TDI; Sigma-Aldrich, Burlington, MA, USA), fumaric acid (FA; Sigma-Aldrich, Burlington, MA, USA), and pentaerythritol triacrylate (PETA; Sigma-Aldrich, Burlington, MA, USA) were used as monomers to design a crosslinkable polyamide-imide (PAX). 6-FDA, FA, and TDI were polymerized at a monomer ratio of 0.9:0.1:1.1 in dimethylacetamide (DMAc; Sigma-Aldrich, Burlington, MA, USA) 100 °C for 1 h. The monomer content in DMAc was 60 wt %. 6-FDA was used to ensure solubility in acetone, and FA and TDI were selected to promote dimensional stability in the electrolyte solution and crosslinking. To improve crosslinking, the residual-NCO group was capped with PETA at a monomer ratio of 6-FDA + FA + TDI:PETA of 1:1, at 70 °C for 1 h. The PAIX was characterized using gel permeation chromatography (GPC; Waters Co., Ltd., Milford, MA, USA) and thermogravimetric analysis (TGA; Discovery TGA; TA Instruments, New Castle, DE, USA).

### 2.2. Polyethylene Separator Coating

A 12-μm-thick polyethylene separator was used as a substrate, with a Gurley number of 120 s/100 cc. The following hybrid organic/inorganic layers with various compositions were deposited on the substrate to determine the adhesion and permeability (Gurley number): polyurethane acrylate, polyvinylidene fluoride (PVdF), Al_2_O_3_ nanoparticles = 0.8, 0.2, 2.0; PAIX, PVdF, Al_2_O_3_ nanoparticles = 1.0, 0, 2.0; PAIX, PVdF, Al_2_O_3_ nanoparticles = 0.9, 0.1, 2.0; PAIX, PVdF, Al_2_O_3_ nanoparticles = 0.8, 0.2, 2.0; PAIX, PVdF, Al_2_O_3_ nanoparticles = 0.7, 0.3, 2.0; PAIX, PVdF, Al_2_O_3_ nanoparticles = 0.8, 0.2, 3.0; and PAIX, PVdF, Al_2_O_3_ nanoparticles = 0.7, 0.3, 3.0. The adhesion strengths of the hybrid coating layers were evaluated by the 180° peel test between the hybrid coating layer and the 3 M adhesive tape (Scotch Magic tape, 18 mm. 3 M) adhered to their surface using a Universal Testing Machine (Instron 3343, Instron, Norwood, MA, USA). Changes in permeability as a result of humidity and nonsolvent effects were examined. Gurley numbers were measured by the JIS P8117 method (Testing Method for Air Permeability of Paper and Paperboard [27]) using a Digital Oken Type Air-Permeability tester (EGO-1-55-1MR, Asahi Seiko Co., Ltd., Osaka, Japan). All coatings were applied and tested using pilot-scale techniques.

### 2.3. Thermal and Electrochemical Characterization

The thermal characteristics of separators coated with ceramic or hybrid organic/inorganic layers were tested by heating the separators at 200 °C for 10 min. Coated separators were then assembled into a cylindrical battery pack filled with electrolyte and heated to 150 °C for 1 h to further evaluate their thermal behavior. Cylindrical mini-18650 cells were assembled in a dry-room, and the rate and cycling performance of the hybrid coated separators were examined. A Li(Ni, Co, Al)O_2_ (NCA) and Li(Ni, Co, Mn)O_2_ (NCM) mixed cathode (NCA/NCM/carbon black/PVdF = 96/1.8/2.2 by weight, loading level = 40.73 mg cm^−2^) and graphite anode (graphite/styrene-butadiene rubber (SBR)/carboxymethylcellulose (CMC) = 97.5/1.5/1.0 by weight, loading level = 20.50 mg cm^−2^), provided by Samsung SDI (South Korea) were used in the electrochemical performance tests. The cathode and anode were cut into 54 mm× 130 mm and 58 mm× 160 mm pieces, respectively. The separator was 60 mm wide. The liquid electrolyte comprised 1.15 M LiPF_6_ in ethylene carbonate (EC), ethyl methyl carbonate (EMC), and dimethyl carbonate (DMC) (EC/EMC/DMC 2/4/4 by volume; PANAX ETEC Co., Busan, Korea) with 1% lithium difluorophosphate (MP1), and 1.5% vinyl chloride (VC) additives. Galvanostatic rate and cycling tests were conducted in the range 3.0–4.25 V under constant current-constant voltage charging and constant current discharging modes (WonATech, Seoul, Korea). The direct current internal resistance (DCIR) of as-assembled cells and cells that had been cycled 100 times was measured. Charge and discharge rates were tested at current density C-rates between 0.1 and 5C. In the cycling tests, the cells were charged to 4.25 V at 1C with a 0.2C current cut-off, and then discharged to 3.0 V at 1C. The cells were left to stabilize for 10 min after charging and 15 min after discharging.

## 3. Results and Discussion

**Figure 1:**
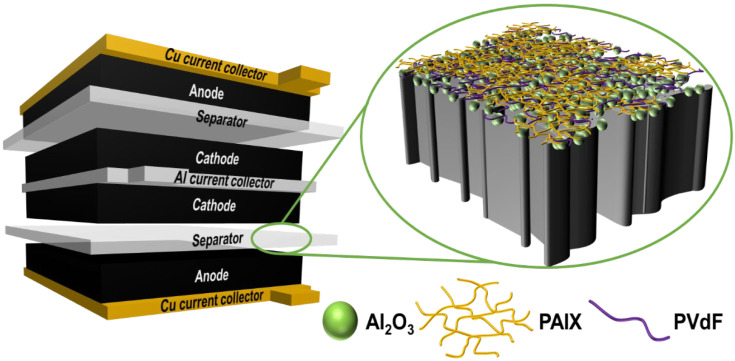
Schematic of a Li rechargeable battery in which the separator is coated with a hybrid organic/inorganic layer.

A separator coated with a hybrid organic/inorganic layer was placed between an NCA cathode and graphite anode; the hybrid layer was in contact with the cathode, as shown in Figure 1. The hybrid layer comprised inorganic Al_2_O_3_ nanoparticles, organic PAIX, and a PVdF binder. The inorganic-coated polyolefin is inexpensive (<$1 m^−2^), dimensionally stable, and highly wettable in carbonate electrolyte, whereas the polymer-coated separator is flexible, forms intimate contact with the electrode, and has better electrolyte uptake [28,29,30,31,32]. However, the inorganic-coated polyolefin is brittle after thermal treatment, and the polymer coating is less robust against mechanical failure modes. Therefore, the hybrid organic/inorganic coating was designed to exploit the advantages and compensate for the disadvantages of each layer component.

**Figure 2:**
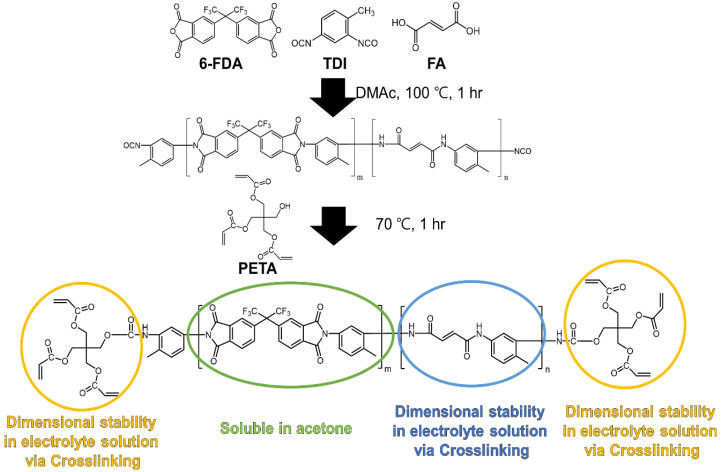
Schematic of PAIX synthesis. 6-FDA, FA, and TDI monomers were polymerized in DMAc at 100 °C for 1 h, and PETA was used as a multifunctional terminal.

A schematic of the PAIX synthesis process is presented in Figure 2. First, 6-FDA, TDI, and FA monomers were polymerized in DMAc at 100 °C for 1 h; then, PETA was added as a multifunctional crosslinker [33,34]. It is important that the components are soluble to ensure processibility; 6-FDA was crosslinked with the TDI using an isocyanate-anhydride reaction [35] so that it was soluble in acetone. The acrylamides, formed from the addition of isocyanate and alcohol [35], were crosslinked with acrylates so that the product was stable in the carbonate electrolyte. After the hybrid organic/inorganic layer was cast on the polyolefin separator, a crosslinking process was initiated using benzoyl peroxide (BPO; 10 wt % with respect to the PAIX content) and left to react overnight at 100 °C.

**Figure 3:**
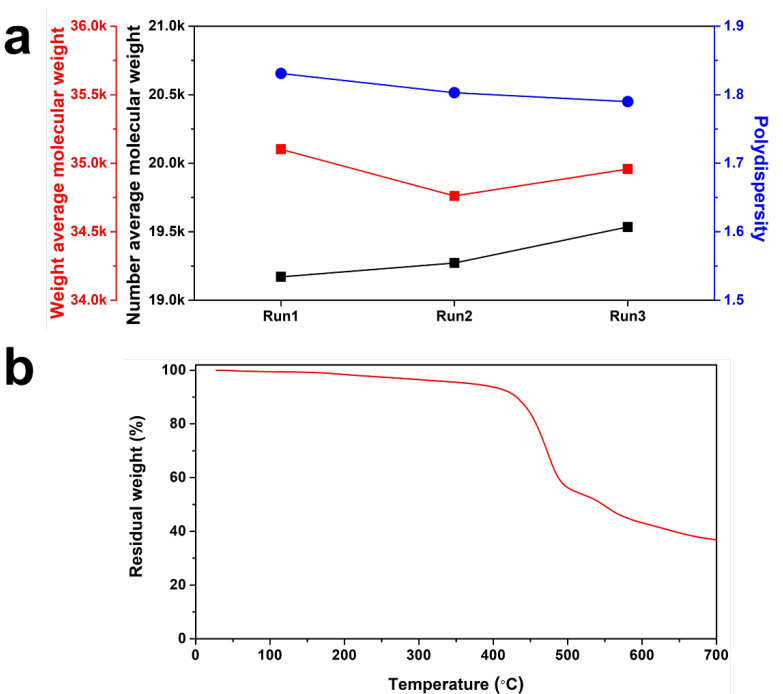
Macromolecular properties of the PAIX. (**a**) GPC and (**b**) TGA results.

The pilot-scale PAIX synthesis process was repeated three times to determine the reproducibility, and its macromolecular properties were studied using GPC and TGA as shown in Figure 3. The PAIX was dissolved in DMF in preparation for GPC testing. As shown in Figure 3a, the number (M_n_) and weight (M_w_) of the average molecular weights were approximately 19,325 and 34,940, respectively. The inter-sample molecular weights differed by <1.1%. The polydispersity index (PDI), defined as M_w_/M_n_, was approximately 1.8, which agrees with the PDI of previous polyamide-imide synthesis works [36]. Therefore, we concluded that the pilot-scale polymerization produced a product of reasonable quality. TGA was used to assess the thermal stability, as shown in Figure 3b. The TGA tests were conducted under a nitrogen blanket between 25 and 700 °C at a ramp rate of 5 °C per minute. Significant weight loss was observed above 400 °C, which coincides with the polyamide-imide decomposition temperature [37]. These results show that the pilot-scale PAIX synthesis was reproducible and thermally stable, and suitable for use as the organic component of the hybrid organic/inorganic coating.

The optimal hybrid coating recipe was analyzed, and the results are presented in Table 1. ASTM test D726 [38,39] was used to measure the permeability of a 12-µm-thick polyethylene separator, which yielded a Gurley number of 120 s/100 cc. The polyethylene separator was coated with a range of test slurries. A mixture of polyurethane acrylate and PVdF was applied to produce an organic reference coating, which is referred to as acrylate in Table 1. The Gurley number of the acrylate sample was 1770 s/100 cc, which is more than 14 times greater than that of the untreated separator material. PAIX 100% was used to create a second organic reference coating, referred to as PAIX12 in Table 1. The Gurley number of the PAIX12 sample was 226 s/100 cc, which is less than twice that of the untreated separator material. The PAIX-polyethylene and acrylate-polyethylene adhesion forces were 49.2 and 193.2 gf cm^−1^, respectively. Commercial cell standards require that the adhesion force between the coating layer and substrate exceed 100 gf cm^−1^; thus, a PVdF binder was used to increase the PAIX coating adhesion without significantly increasing the permeability.

PVdF binder was added to PAIX to produce PAIX1291 and PAIX1282 with adhesion forces of 206.1 and 226.4 gf cm^−1^, respectively, but the Gurley numbers increased to 582 and 820 s/100 cc, respectively. The Gurley number was reduced by decreasing the organic:Al_2_O_3_ ratio from 1/2 to 1/3. Consequently, the Gurley number of PAIX1382 was 326 s/100 cc with an adhesion force of 104.8 gf cm^−1^. Finally, the recipe was tuned by adding more PVdF binder and increasing the solid content to 12 wt %, to produce PAIX1373-12 with a Gurley number of 278 s/100 cc.

**Figure 4:**
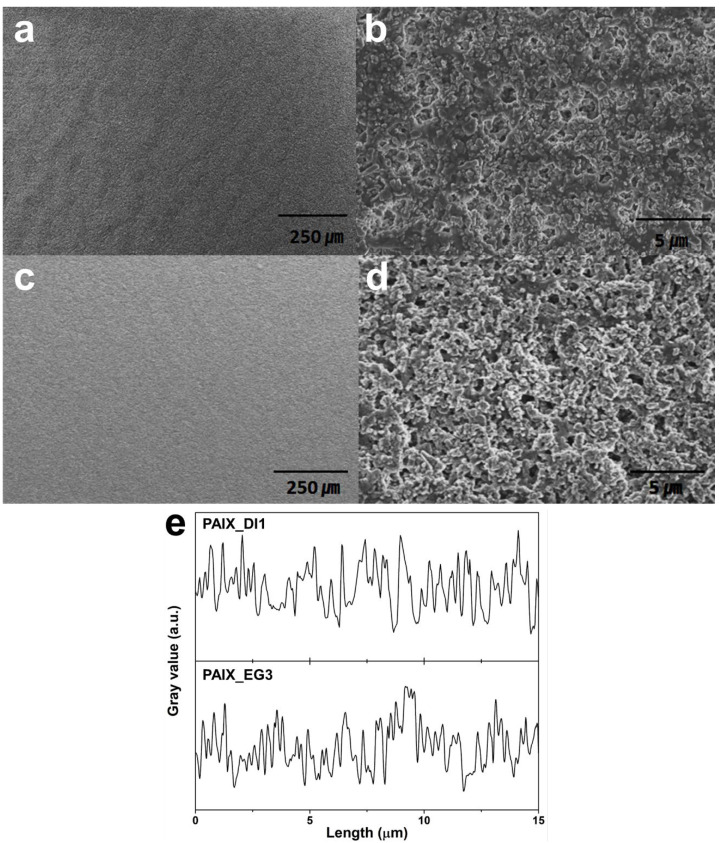
Hybrid organic/inorganic coating morphologies. (**a**) Low- and (**b**) high-magnification SEM images of a PAIX-PVdF-Al_2_O_3_ layer with a 1 wt % distilled water additive (PAIX_DI1). (**c**) Low- and (**d**) high-magnification SEM images of a PAIX-PVdF-Al_2_O_3_ layer with a 3 wt % ethylene glycol additive (PAIX_EG3). (**e**) Surface roughness of the PAIX_DI1 and PAIX_EG3 samples.

Nonsolvent additives were introduced into the optimized PAIX1373-12 recipe to increase the permeability, as shown in Table 2. The coating thickness and density, and the organic:Al_2_O_3_ and PAIX:PVdF ratios, were precisely controlled, and the phase separation effect was used to decrease the coating density by adding distilled water at concentrations of 1 and 2 wt % to produce PAIX_DI1 and PAIX_DI2, respectively. Although PAIX_DI1 and PAIX_DI2 were thicker coatings, the Gurley number decreased to 260 and 245 s/100 cc, respectively. The density was decreased further (2.04 g cc^−1^) by adding ethylene glycol at a concentration of 3 wt % to produce PAIX_EG3 with a Gurley number of 207 s/100 cc, which also mitigated the phase separation and improved the coating uniformity. The morphologies of PAIX_DI1 and PAIX_EG3 are given in Figure 4. Low-magnification SEM images of PAIX_DI1 and PAIX_EG3 suggest that the coating surfaces are uniform, but high-magnification images reveal the surface roughness that is introduced by phase separation. The surface of PAIX_DI1, shown in Figure 4b, was significantly rougher than the surface of PAIX_EG3, shown in Figure 4d. As presented in Figure 4e, the PAIX_DI1 and PAIX_EG3 surfaces featured an average of 2.27 and 3.00 peaks per unit micrometer, respectively. Therefore, we concluded that ethylene glycol was the best nonsolvent to form a highly permeable hybrid organic/inorganic coating layer.

**Figure 5:**
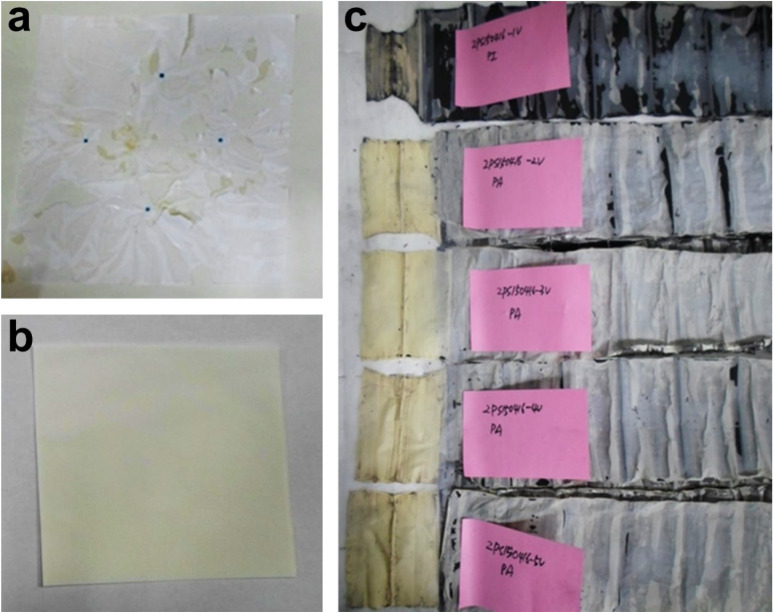
Photographs of separator samples following dry and wet thermal tests. (**a**) Acrylate- and (**b**) hybrid PAIX/AL_2_O_3_-coated separators after dry thermal treatment at 200 °C for 10 min, and (**c**) disassembled acrylate- (top) and hybrid PAIX/Al_2_O_3_-coated (second to fifth) separators after immersion in an electrolyte solution at 150 °C for 1 h.

Polyolefin becomes deformable below 180 °C [40,41,42] so it is important that the hybrid coating protects the separator. The thermal durability of the PAIX coating was tested using dry and wet thermal exposure tests, as shown in Figure 5. The hybrid polyurethane acrylate/Al_2_O_3_ (organic:Al_2_O_3_ ratio of 1/8 and Gurley number of 145 s/100 cc)—coated reference separator was severely damaged by the dry exposure test, as shown in Figure 5a, and the separator coated with PAIX/Al_2_O_3_ was preserved without shrinkage after the same test, as shown in Figure 5b. The yellow color visible in the images was a result of imidization during thermal exposure. Separator samples were immersed in an electrolyte solution at 150 °C for 1 h and then disassembled and photographed, as shown in Figure 5c. The separator coated with polyurethane acrylate/Al_2_O_3_ shrank significantly during the test, whereas the hybrid PAIX/Al_2_O_3_ coating resisted deformation during exposure to the same conditions. The enhanced thermal properties of the hybrid PAIX/Al_2_O_3_ layer were clearly demonstrated by these tests, which show that the hybrid coating was more thermally robust than the untreated and acrylate-coated separators.

**Figure 6:**
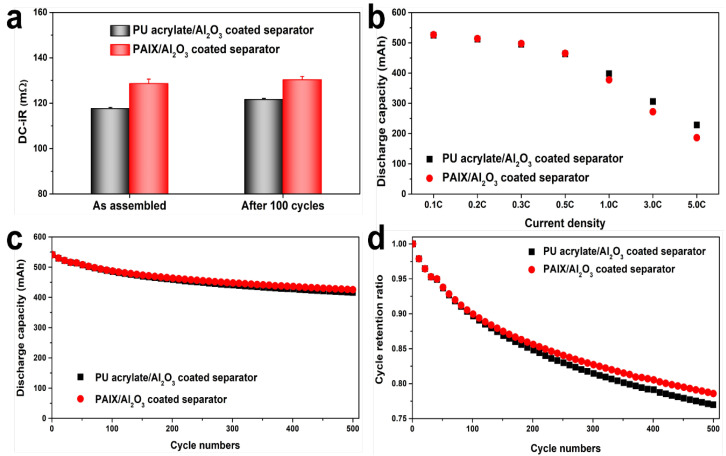
Electrochemical performance tests. (**a**) The DCIR increase of the cells, as-assembled and after 100 cycles. (**b**) The rate performance, (**c**) cycling discharge capacity, and (**d**) cycle retention ratio at 1.0C for each cell design.

The electrochemical performance of the separator with a hybrid organic/inorganic layer was tested by incorporating it into cylindrical mini-18650 cell assemblies. As shown in Figure 6a, the average DCIR of the as-assembled reference cell with an acrylate-coated separator was 117.6 mΩ and the DCIR of the separator with a hybrid PAIX coating was 128.6 mΩ. The impedance of the PAIX-based cell was greater than that of the reference cell because of its relatively low permeability. However, the structural stability of the PAIX-based separator meant that the DCIR value increased by < 1.7 mΩ after 100 charge cycles, whereas the reference cell DCIR increased by 4 mΩ. Figure 6b shows that the discharge capacity of the cells was similar at lower rates (≤ 0.5C), but the capacity of the PAIX cell was lower than that of the reference cell at higher discharge rates. At 1.0, 3.0, and 5.0C, the PAIX cell discharge capacity was 94.9%, 89.0%, and 81.5% of the reference cell capacity, respectively. This is because of the higher DCIR of the PAIX cells. Figure 6c,d present the cycling performance of the reference and PAIX cells, respectively. The cells were cycled 500 times each at 1.0C, after which the better cyclability of the PAIX cell was clear. The discharge capacities of the reference and PAIX cells after 500 cycles were 416.3 and 425.5 mAh, respectively, which were 77.0% and 78.6% of the initial discharge capacities, respectively. These tests suggest that although the hybrid PAIX/Al_2_O_3_ coating worsens electrolyte permeability, the negative effects are less significant at low current densities, and the performance improves over many cycles. Based on the material properties and electrochemical performances, it was summarized as follows: i) the electrochemical performances of the PAIX cells at low rates (≤ 0.5C) were comparable to the that of the reference cell, ii) the discharge capacities of the PAIX cells at high rates were inferior to the reference cells due to the lower permeability, and iii) the discharge capacities of the PAIX cells after 500 cycles were higher than that of the reference cells owing to the improved structural stability. Therefore, we conclude that a separator coated with a hybrid PAIX/Al_2_O_3_ layer has a minor negative impact on the cell rate performance, but improves the thermal stability at high temperatures even when the cell is filled with electrolyte.

## 4. Conclusions

A pilot-scale method for synthesizing a hybrid organic/inorganic coating was developed from a crosslinkable polymer synthesis technique and optimized. Multistep polymerization was used to successfully synthesize a reproducible, thermally stable PAIX with high thermal decomposition temperatures. The PAIX and PVdF ratio, organic to Al_2_O_3_ ratio, solid content, and nonsolvent additive concentration were controlled and optimized to produce a thin (≤4 μm), permeable (≤250 s/100 cc), and thermally stable (>150 °C) hybrid coating. The reduced permeability owing to the hybrid coating layer caused the DCIR to increase, and the discharge capacity to decrease at high rates (>1.0 C). However, in comparison with reference cells, mini-18560 cells that incorporated the hybrid separator coating showed the same discharge capacity at low rates and better cycling retention after 500 charge cycles. Therefore, we conclude that a polyolefin separator coated with a hybrid organic PAIX and inorganic Al_2_O_3_ layer provides a pathway to more thermally stable commercial Li-ion batteries with few electrochemical performance sacrifices.

## Figures and Tables

**Table 1:** Coating recipes tested and corresponding Gurley numbers.

Sample Code	Solid Content (wt %)	Organic to Al_2_O_3_ Ratio	Organic Content (wt %)			Gurley Number (s/ 100 cc)
			PAIX	Polyurethane Acrylate	PVdF	
Acrylate	10	1/2	0	80	20	1770
PAIX12	10	1/2	100	0	0	226
PAIX1291	10	1/2	90	0	10	582
PAIX1282	10	1/2	80	0	20	820
PAIX1382	10	1/3	80	0	20	326
PAIX1373-12	12	1/3	70	0	30	278

**Table 2:** Summary of the hybrid coating layer properties with the addition of nonsolvents.

Sample Code	Solid Content (wt %)	Organic to Al_2_O_3_ Ratio	PAIX to PVdF Ratio	Density (g cc^−1^)	Thickness (μm)	Gurley Number (s/100 cc)
PAIX1373-12	12	1/3	7/3	2.15	2.500	278
PAIX_DI1				1.84	3.000	260
PAIX_DI2				1.48	3.875	245
PAIX_EG3				2.04	4.000	207

## Data Availability

Not applicable.
